# Ursodeoxycholic acid improves liver function via phenylalanine/tyrosine pathway and microbiome remodelling in patients with liver dysfunction

**DOI:** 10.1038/s41598-018-30349-1

**Published:** 2018-08-08

**Authors:** Da Jung Kim, Seonghae Yoon, Sang Chun Ji, Jinho Yang, Yoon-Keun Kim, SeungHwan Lee, Kyung-Sang Yu, In-Jin Jang, Jae-Yong Chung, Joo-Youn Cho

**Affiliations:** 1 Department of Clinical Pharmacology and Therapeutics, Seoul National University College of Medicine and Hospital, Seoul, Korea; 2 Department of Clinical Pharmacology and Therapeutics, Seoul National University College of Medicine and Bundang Hospital, Seongnam, Korea; 3 MD Healthcare Inc., Seoul, Korea; 4 Metabolomics Medical Research Center, Seoul National University College of Medicine, Seoul, Korea

**Keywords:** Metabolomics, Translational research

## Abstract

Ursodeoxycholic acid (UDCA) is a metabolic by-product of intestinal bacteria, showing hepatoprotective effects. However, its underlying molecular mechanisms remain unclear. The purpose of this study was to elucidate the action mechanisms underlying the protective effects of UDCA and vitamin E against liver dysfunction using metabolomics and metagenomic analysis. In this study, we analysed blood and urine samples from patients with obesity and liver dysfunction. Nine patients were randomly assigned to receive UDCA (300 mg twice daily), and 10 subjects received vitamin E (400 IU twice daily) for 8 weeks. UDCA significantly improved the liver function scores after 4 weeks of treatment and effectively reduced hepatic deoxycholic acid and serum microRNA-122 levels. To better understand its protective mechanism, a global metabolomics study was conducted, and we found that UDCA regulated uremic toxins (hippuric acid, *p*-cresol sulphate, and indole-derived metabolites), antioxidants (ascorbate sulphate and *N*-acetyl-L-cysteine), and the phenylalanine/tyrosine pathway. Furthermore, microbiome involvement, particularly of *Lactobacillus* and *Bifidobacterium*, was demonstrated through metagenomic analysis of bacteria-derived extracellular vesicles. Meanwhile, vitamin E treatment did not result in such alterations, except that it reduced uremic toxins and liver dysfunction. Our findings suggested that both treatments were effective in improving liver function, albeit via different mechanisms.

## Introduction

Non-alcoholic fatty liver disease (NAFLD) is diagnosed when more than 5% of hepatocytes are steatotic in patients who do not consume excessive alcohol, and the disease severity ranges from simple steatosis and steatohepatitis to advanced fibrosis and cirrhosis^1^. The prevalence of NAFLD is currently estimated to be 24% in the United States^2^. Physical exercise and weight loss are common therapeutic strategies for NAFLD; however, treatments based on lifestyle changes often fail, thus necessitating drug therapy.

Ursodeoxycholic acid (UDCA), a secondary bile acid produced by intestinal bacteria as a metabolic by-product, has been shown to be effective in the nonsurgical treatment of cholesterol gallstones and primary biliary cirrhosis (PBC). A recent study demonstrated that 2 years of UDCA (600 mg/day) or vitamin E (800 IU/day) treatment effectively reduced liver dysfunction in Indian NAFLD patients^3^. However, the efficacy of UDCA is still controversial^3,4^, and no metabolic profiling studies have been conducted to reveal the therapeutic effects of UDCA in liver disease.

Global metabolomics is a promising approach for understanding disease mechanisms and for biomarker discovery. It has been extensively used in the diagnosis and monitoring of disease progression, providing crucial insights into disease pathogenesis. Particularly in the case of liver diseases, global metabolomics has been applied to the development of diagnostic or prognostic biomarkers. Yamakado *et al*.^5^ have identified a set of biomarkers, typically indicating alterations in serum aromatic amino acids (AAAs) and branched amino acids (BAAs), to classify NAFLD into liver inflammation and fibrosis stages. These findings strongly indicate that a metabolic imbalance causes NAFLD.

In the current study, targeted and global metabolomic analyses were carried out to identify significantly changed endogenous metabolites, including bile acids, which may be related to the mechanisms underlying the protective effects of UDCA and vitamin E against liver dysfunction. Further, metagenomic analysis was used to find potential links between bacteria-derived metabolites, discovered by global metabolomics, and drug responses. This study reveals how UDCA and vitamin E treatments influence bile acid levels, metabolomic profiles, microRNA (miRNA) levels, and the intestinal microbiome composition to exert hepatoprotective effects.

## Results

### UDCA and vitamin E improved liver function

**Figure 1:**
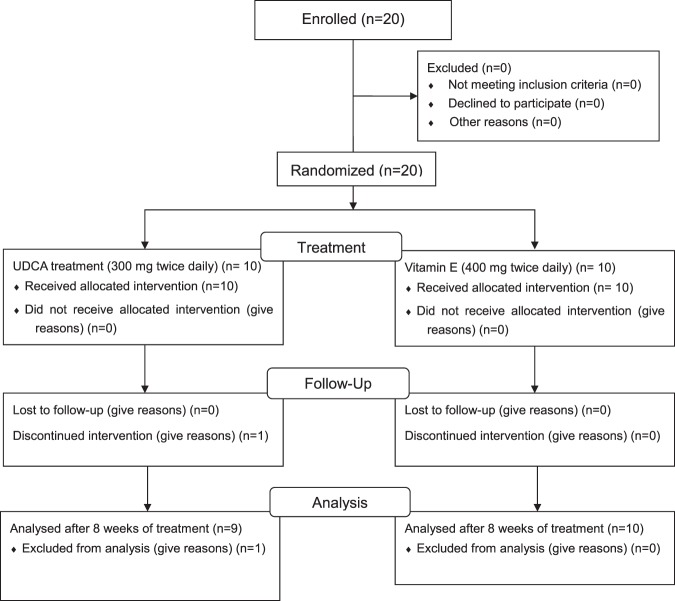
Flow chart of the study. A total of 20 subjects were enrolled in this study and underwent randomisation. Ten of them were assigned to take UDCA (300 mg twice daily), and the other 10 were treated with vitamin E (400 IU twice daily) for 8 weeks. During this period, one subject withdrew from the UDCA group for a personal reason after 4 weeks and therefore, was excluded from data analysis. The total number of subjects included in the final analysis was nine for the UDCA group and 10 for the vitamin E group.

Flow diagram of the experimental procedure is shown in Fig. 1. To determine the efficacy of the drugs, we measured liver function parameters in the serum, and the results for each group are listed in Table 1. Compared with their baseline values, the alanine aminotransferase (ALT), aspartate aminotransferase (AST), and gamma-glutamyl transferase (GGT) levels were reduced by 40.3%, 33.9%, and 23%, respectively, after 4 weeks of UDCA treatment. Reduced levels were maintained at week 8, but the difference with the baseline was only significant for GGT, probably because of variations associated with the small sample size. Vitamin E treatment was also effective in improving the ALT and AST levels at both week 4 and week 8, while the GGT level was only reduced at week 8.

**Table 1:** Liver function parameters measured in the serum before and after treatment.

Liver function parameters	UDCA (n = 9)				Vitamin E (n = 10)			
	Pre-dose	Week 4	Week 8	P-value	Pre-dose	Week 4	Week 8	P-value
Alanine aminotransferase (IU/L)	81.56 ± 11.63	48.67 ± 8.07	57.67 ± 12.56	^†^	80.7 ± 9.5	56.6 ± 10.1	50 ± 9	†,‡
Aspartate aminotransferase (IU/L)	42.22 ± 4.24	27.89 ± 3.49	35.22 ± 7.59	^†^	45.1 ± 6.3	33.7 ± 5.5	30.9 ± 3.6	†,‡
Gamma-glutamyl transferase (IU/L)	87 ± 18	66.56 ± 12.59	64.11 ± 12.01	^†,‡^	60.6 ± 14.2	47.1 ± 7.9	47.1 ± 9.0	‡
miR-122 (fold change)	1.00 ± 0.00	0.6078 ± 0.5740	0.5733 ± 0.6330	^†,‡^	1.00 ± 0.00	0.738 ± 0.514	1.073 ± 0.933	ns

Data are the mean ± standard deviation. ^†^P < 0.05 (Friedman test) between pre-dose and week 4; ^‡^P < 0.05 between pre-dose and week 8. ns, not significant.

### UDCA reduced miR-122 expression

Circulating miR-122 is considered as  a potential biomarker for liver disease^6^. It is the most frequently detected miRNA in the adult liver, accounting for approximately 70% of the total liver miRNA population^7^ and controlling liver-enriched transcription factors, including hepatocyte nuclear factor-4α^6^ and p53^8^, thereby maintaining the proper balance between proliferation and differentiation of hepatocytes. It has been reported that miR-122 is upregulated in patients with hepatitis C and NAFLD^6^.

Consistent with previous studies, the miR-122 level was significantly reduced by UDCA treatment (Table 1), by 39% at week 4 and by 42% at week 8. These results suggested that UDCA might protect hepatocytes by suppressing miR-122. Interestingly, vitamin E did not significantly reduce the miR-122 level at both time points, probably because we did not exclude outliers. At week 8, one subject in the vitamin E group had an approximately two-fold higher level of miR-122 than those at pre-dose and week 4, and another subject had a more than three-fold higher miR-122 level at week 8 (data not shown). Nevertheless, vitamin E treatment was also effective in reducing liver dysfunction (Table 1).

### UDCA only decreased hydrophobic bile acids

**Figure 2:**
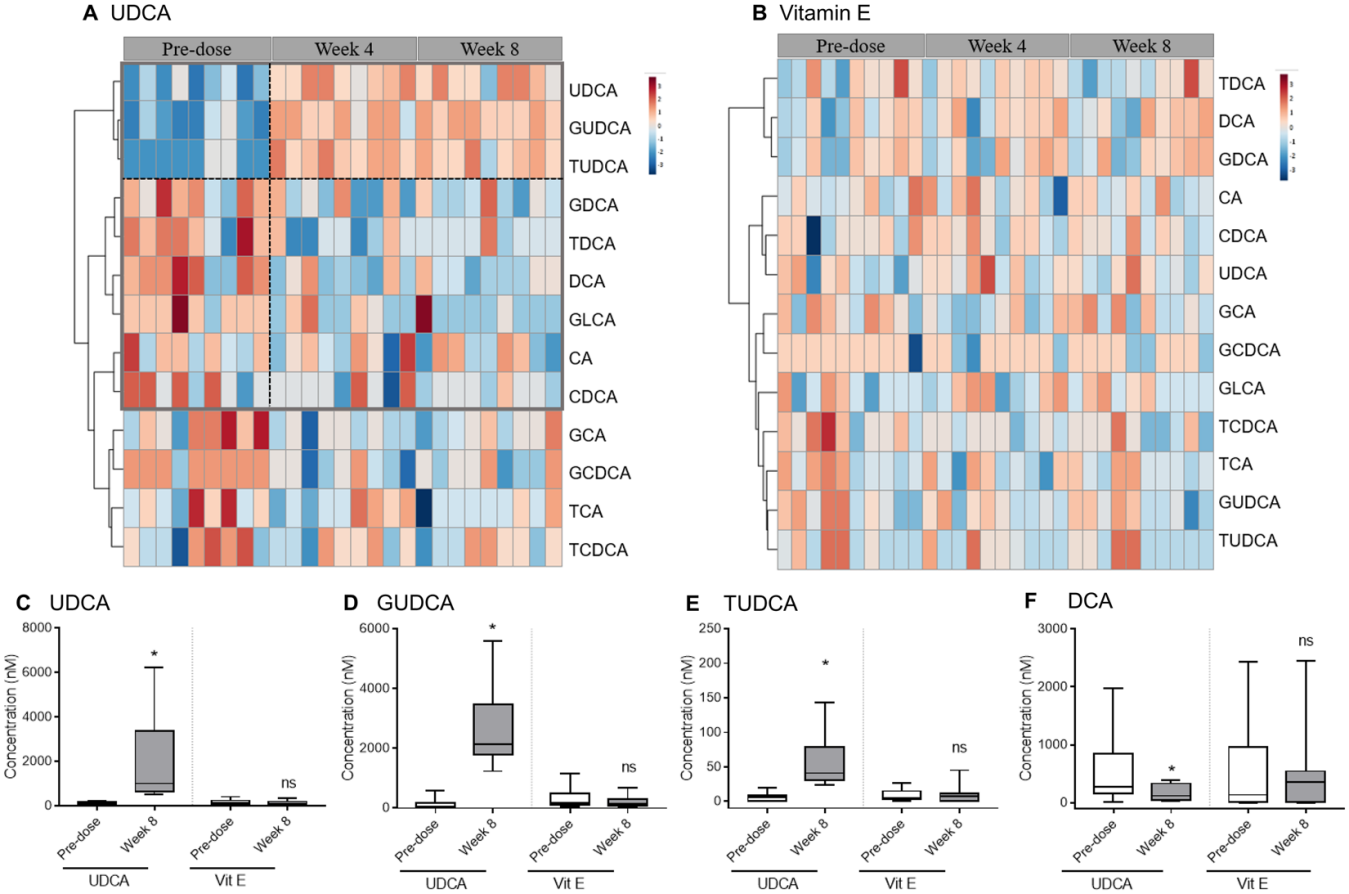
Heatmaps and boxplots of bile acids that significantly changed after treatments. For heatmaps, the plasma concentration of each bile acid was converted to a log scale. Normalisation was carried out using the mean value of each bile acid. Heatmaps show changes in bile acid levels from pre-dose to week 4 and week 8 for (**A**) UDCA and (**B**) vitamin E treatments. The colour scale ranges from −3 (blue) to 3 (red). Dotted lines distinguish the clusters of bile acids showing distinct patterns after treatment compared with those at pre-dose. Boxplots represent the concentrations (nM) of each bile acid: (**C**) UDCA, (**D**) GUDCA, (**E**) TUDCA, and (**F**) DCA. The boxplots show 5% and 95% confidence intervals. A paired *t*-test was used for statistical evaluation. *P < 0.05 between pre-dose and week 8; ns, non-significant difference between the groups.

A total of 15 bile acids, including primary and secondary bile acids, were measured in the plasma. Lithocholic acid (LCA) and taurolithocholic acid were excluded from analysis because their levels were below the detection limit. A heatmap was plotted by converting changes in bile acid concentrations, compared with their pre-dose concentrations, into a log scale (Fig. 2A,B). The plasma levels of bile acids, particularly UDCA and its conjugates, tauroursodeoxycholic acid (TUDCA) and glycoursodeoxycholic acid (GUDCA), were elevated at weeks 4 and 8 of UDCA treatment (Fig. 2C–E). The level of deoxycholic acid (DCA) was statistically significantly reduced (Fig. 2F). The decreases in the levels of DCA conjugates (glycodeoxycholic acid and taurodeoxycholic acid) and glycolithocholic acid were not significant. Raw values of concentrations (non-scaled values) were used for statistical comparisons. After vitamin E treatment, none of the bile acid levels changed during the study. There was no exclusion of outliers nor any correction for baseline levels across all the subjects because of the small sample size. In summary, UDCA only reduced hydrophobic bile acids, especially DCA, in subjects with liver dysfunction.

### Principal component analysis revealed clustering of samples based on UDCA and vitamin E treatments

**Figure 3:**
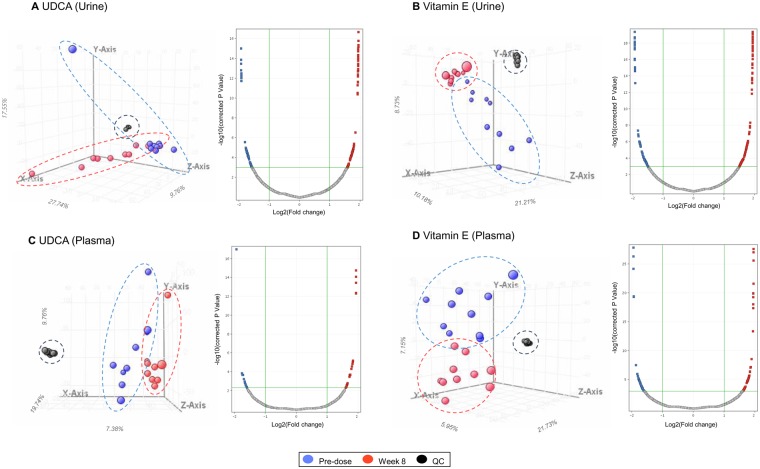
Estimation of urinary and plasma metabolites after UDCA and vitamin E treatments via global metabolomic analysis. Three-dimensional PCA data and volcano plots are shown for urine samples of (**A**) UDCA- and (**B**) vitamin E-treated patients and plasma samples of (**C**) UDCA- and (**D**) vitamin E-treated patients. In the PCA plot, blue and red spheres indicate pre-dose and week 8 samples, respectively. Black spheres represent the quality control sample used to verify the reproducibility and accuracy of the data. Each dotted circle indicates a cluster of spheres with the same colour. Values in the volcano plots show the metabolites that significantly changed, as found by multivariate analysis. Red squares indicate metabolites upregulated after treatment, and blue squares indicate metabolites downregulated after treatment.

To assess systemically changed small-molecule metabolites after each drug treatment, we conducted global metabolomic analysis. The volcano plots presented in Fig. 3 show the most significant metabolites found by univariate analysis. Three-dimensional principal component analysis (PCA) score plots showed clear differences between the two time points of each treatment. For example, Fig. 3A demonstrates that the red spheres (week 8) were separated from the blue spheres (pre-dose), showing different metabolome signatures between the groups, with the overall accuracies of 55.1% for urine samples (Fig. 3A) and 36.9% for plasma samples (Fig. 3C) in the UDCA group. Metabolomic profiling also revealed a clear distinction between pre-dose and post-dose values after vitamin E treatment, with the overall accuracies of 40.1% in the urine (Fig. 3B) and 40.8% in the plasma (Fig. 3D).

### UDCA and vitamin E changed aromatic amino acids and their metabolites

**Figure 4:**
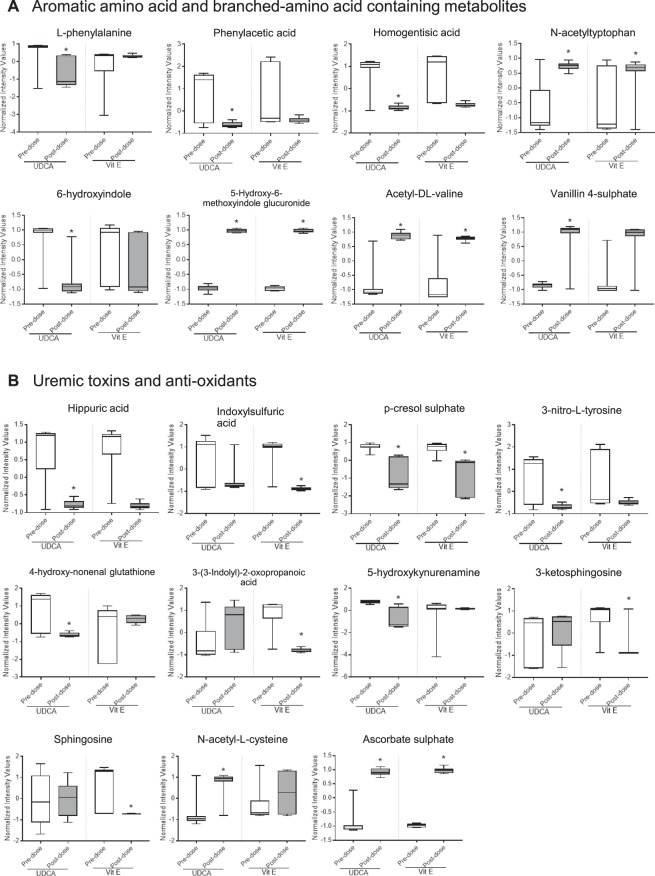
Relative intensity of identified metabolites. Relative intensities of each identified metabolite in the urine and plasma are shown with boxplots. (**A**) Metabolites containing aromatic amino acids or branched amino acids (L-phenylalanine, phenylacetic acid, homogentisic acid, *N*-acetyltryptophan, 6-hydroxyindole, 5-hydroxy-6-methoxyindole glucuronide, acetyl-DL-valine, and vanillic acid sulphate). (**B**) Metabolites known to be toxic [hippuric acid, indoxyl sulphuric acid, *p*-cresol sulphate, 3-nitro-L-tyrosine, 4-hydroxynonenal glutathione, 3-(3-indolyl)-2-oxopropanoic acid, 5-hydroxykynurenamine, 3-ketosphingosine, and sphingosine] or antioxidants (*N*-acetyl-L-cysteine and ascorbate sulphate). The boxplots show 5% and 95% confidence intervals. A paired *t*-test was used for statistical evaluation. *FDR-adjusted P-value < 0.05 between pre-dose and week 8.

The metabolites identified by multivariate analysis were selected according to their Q-values, which are P-values adjusted for the false discovery rate (FDR). The metabolites that showed statistical significance (Q < 0.05) are listed in Supplementary Tables S1 and S2. Figure 4 shows the most frequent small-molecule metabolites produced via the phenylalanine/tyrosine or tryptophan pathway. Typically, as shown in Fig. 4A, L-phenylalanine and its downstream molecules, such as phenylacetic acid and homogentisic acid, were significantly reduced by UDCA. On the contrary, the level of a tryptophan metabolite, *N*-acetyltryptophan, increased. The level of 6-hydroxyindole decreased, and its glucuronidation increased. These data suggest that UDCA reduces systemic indole levels via hydroxylation and glucuronidation. In contrast, vitamin E treatment did not markedly reduce the levels of phenylalanine or indole-derived compounds, except 5-hydroxy-6-methoxyindole glucuronide. Further, induction of BAA-derived metabolites, acetyl-DL-valine and vanillic acid sulphate, supported our hypothesis that UDCA regulates AAAs and BAAs in liver dysfunction.

### UDCA and vitamin E regulated uremic toxins and antioxidants

Uremic toxins such as hippuric acid and *p*-cresol are also products of the phenylalanine/tyrosine pathway, and their levels were well controlled by UDCA (Fig. 4B). Vitamin E only regulated the levels of tryptophan-derived metabolites, indoxyl sulphuric acid and 3-(3-indolyl)-2-oxopropanoic acid. This indicates that UDCA and vitamin E act via distinct mechanisms to exert hepatoprotection. In addition, UDCA upregulated the antioxidants, *N*-acetyl-L-cysteine and ascorbate sulphate, and downregulated the oxidative stressors, 3-nitro-L-tyrosine and 4-hydroxynonenal glutathione (4-HNE GSH). Vitamin E significantly increased the ascorbate sulphate content and decreased inflammatory lipids such as 3-ketosphingosine and sphingosine. Using global metabolomic analysis, we estimated that a number of metabolites with toxic effects or derived from the phenylalanine/tyrosine pathway were less likely to be affected by vitamin E. However, UDCA alleviated the metabolic changes associated with the phenylalanine, tyrosine, and tryptophan pathways and increased the levels of antioxidants in patients with liver dysfunction. A schematic diagram in Fig. 7 shows the metabolic pathways modulated by UDCA treatment.

### Changes in the microbial composition upon UDCA or vitamin E treatment

**Figure 5:**
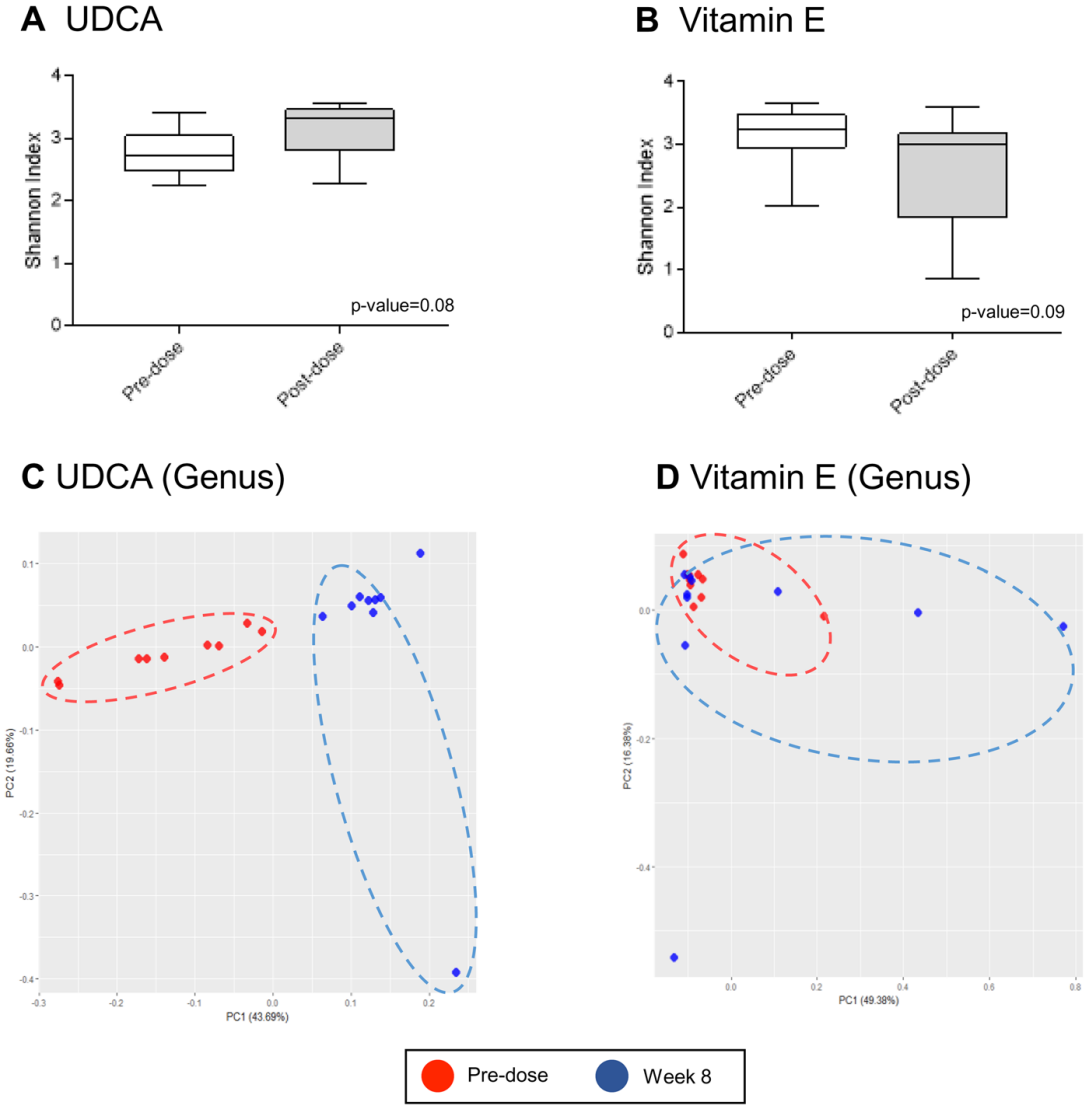
Alpha and beta diversity comparisons of microbiomes collected before and after UDCA treatment. Analyses were performed using sequencing data for the 16S rDNA V3 and V4 regions, with a rarefaction depth of 10,000 reads per sample. Whiskers in the boxplots represent the range of the minimum and maximum alpha diversity values within a population, excluding outliers. Alpha diversity was lower in EV samples collected before the treatment than in samples collected after the treatment. (**A**,**B**) Boxplots of the Shannon index for the UDCA and vitamin E groups, respectively. (**C**,**D**) Principal coordinate analysis of the UDCA and vitamin E treatment data at the genus level, respectively. The proportion of variance explained by each principal coordinate axis is denoted in the corresponding axis label. Red and blue circles indicate samples at pre-dose and week 8, respectively.

**Figure 6:**
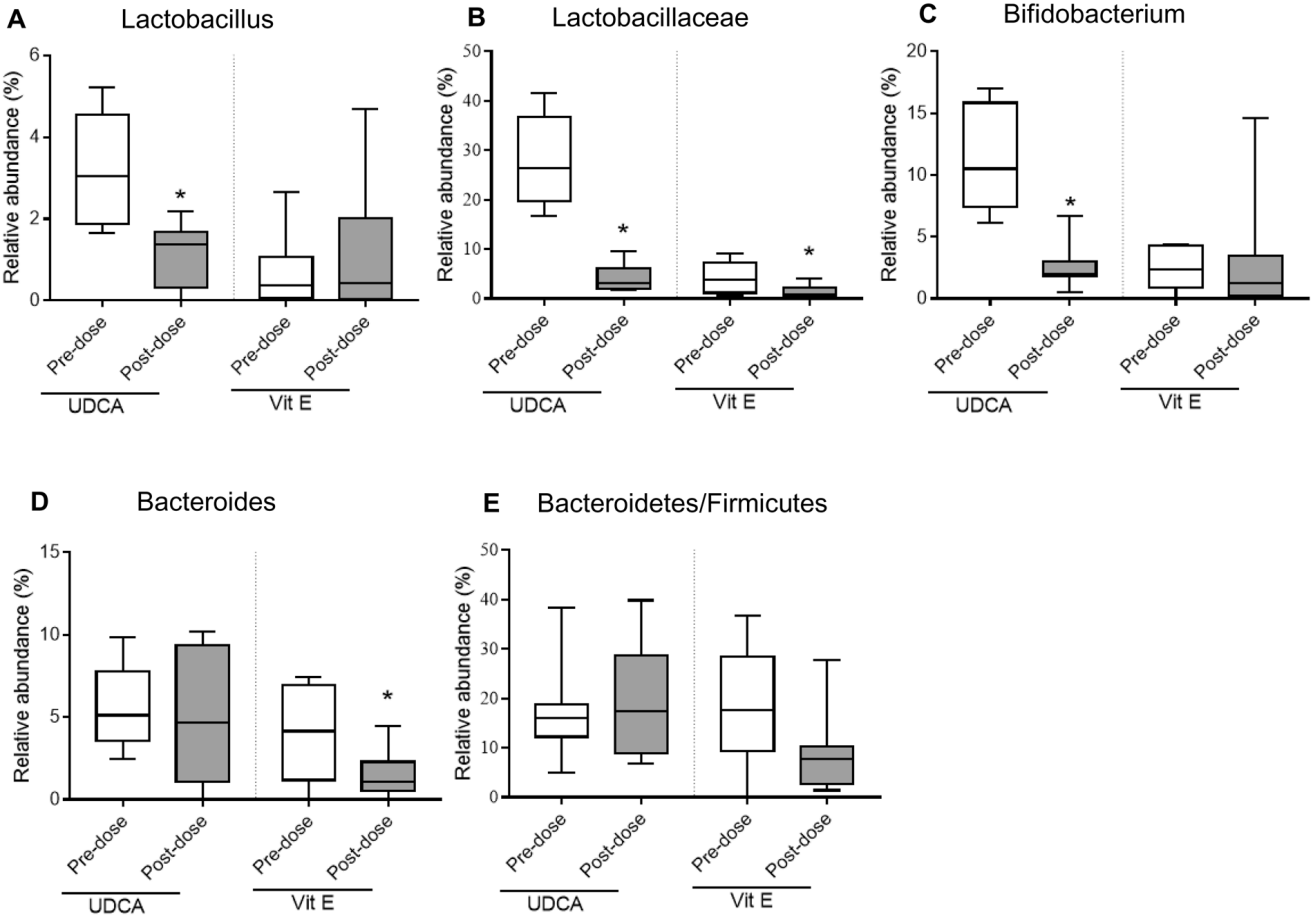
Changes in microbial compositions upon UDCA and vitamin E treatments. Relative bacterial abundances in the microbiome that distinctively changed after 8 weeks of UDCA or vitamin E treatment are shown in bar graphs. (**A**) *Lactobacillus*, (**B**) *Lactobacillaceae*, (**C**) *Bifidobacterium*, (**D**) *Bacteroides*, and (**E**) *Bacteroidetes/Firmicutes* ratio. The results are expressed as the mean ± standard deviation. *P < 0.05 between pre-dose and week 8.

**Figure 7:**
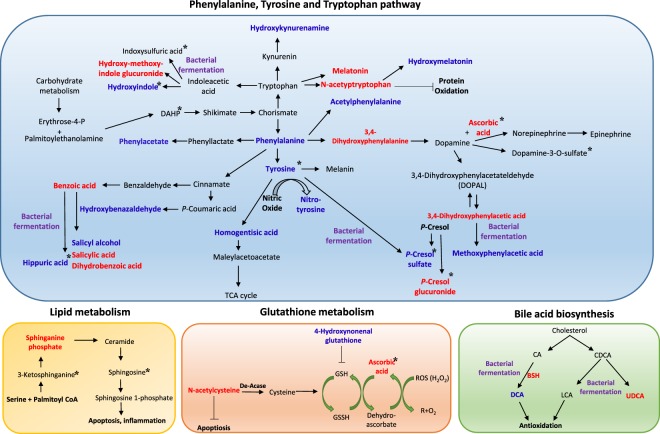
Schematic diagram of potential therapeutic mechanisms of UDCA treatment. Metabolomic analysis revealed that UDCA reduces major compounds in the phenylalanine/tyrosine and tryptophan pathways, including phenylalanine, phenylacetate, acetylphenylalanine, 3,4-hydroxyphenylacetate aldehyde, dopamine-3-*O*-sulphate, hydroxybenzaldehyde, *p*-cresol sulphate, hydroxykynurenamine, hydroxyindole, and hippuric acid, in the plasma and urine. Intermediate metabolites of aromatic amino acids such as hydroxymelatonin, benzoic acid, and salicylic acid, were enhanced. Strong antioxidants such as ascorbate, acetyltryptophan, and *N*-acetyl-L-cysteine were elevated. Further, detoxification of uremic toxins by glucuronidation (hydroxymethoxyindole glucuronide and *p*-cresol glucuronide) was observed after UDCA treatment. However, vitamin E reduced indole propionic acid, indoxyl sulphate, 3-ketosphinganine, and sphingosine, which were not regulated by UDCA. Blue colour indicates a decrease in the metabolite level, and red colour indicates an increase in the metabolite level after UDCA treatment. Metabolites that changed after vitamin E treatment are marked with an asterisk (*). Metabolites that were possibly regulated by bacterial modification are marked with a purple colour.

Global metabolomic profiling revealed that UDCA changed microbe-generated metabolites, such as phenylalanine, methoxyphenylacetic acid, hippuric acid, *p*-cresol, and *N*-acetyltryptophan. This suggested that the microbiota might be involved in bile acid regulation. To evaluate the relationship between microbial composition and responses to UDCA, we conducted a metagenomic analysis using 16S ribosomal DNA (rDNA) sequencing before and after 8 weeks of each treatment. The composition of bacterial microbiota was investigated at the phylum and genus level by metagenomic analysis of bacteria-derived extracellular vesicles (EVs). Comparison of the alpha diversity in the UDCA and vitamin E treatment groups revealed no significant differences based on the Shannon index, as shown in Fig. 5A,B. However, the Chao1 diversity index was lower at pre-dose than at week 8 in both UDCA and vitamin E treatment groups (Supplementary Fig. S1A,B). In Fig. 5C and Supplementary Fig. S1C, the beta diversity in the UDCA group shows clear separation at both phylum and genus levels. In the vitamin E group, however, the results appeared to be poorly clustered before and after 8 weeks of treatment at both levels (Fig. 5D and Supplementary Fig. S1D). The bar graphs of the 16S rDNA sequencing data indicate that UDCA and vitamin E significantly altered the microbial composition after 8 weeks of treatment (Fig. 6). The relative abundance of *Bifidobacterium* markedly declined, by 77.5%, after UDCA treatment for 8 weeks, followed by *Lactobacillus* (64.8%) and *Lactobacillaceae* (85.7%), including all genera (Fig. 6A–C). These results are consistent with those of previous reports on the alteration of intestinal microbiota, including *Bifidobacterium* and *Lactobacillus*, in obese individuals^9–11^. Because the *Bacteroidetes*/*Firmicutes* ratio has been shown to be indicative of obesity *in vivo*^12–14^, we also determined this ratio (Fig. 6E); however, there were no significant changes after UDCA treatment. Vitamin E treatment also changed the composition of certain bacteria. The greatest change in the relative abundance was shown for *Bacteroides*, in which case the relative abundance was reduced by 61.5% compared with that at pre-dose (Fig. 6D). Similar to UDCA treatment, vitamin E treatment reduced the *Lactobacillaceae* abundance (Fig. 6B). Interestingly, the ratio of *Bacteriodetes/Firmicutes* was lowered by vitamin E treatment (Fig. 6E). Further detailed microbial composition data at the phylum and genus levels are reported in Supplementary Fig. S2. Possible pathways involving bacterial modification are indicated in Fig. 7.

## Discussion

In this study, we found that UDCA regulated metabolic pathways (mainly the phenylalanine/tyrosine pathway), reduced the miR-122 expression level, remodelled the microbiome, and changed the bile acid content, thus exerting protective effects on liver function. A potent antioxidant, vitamin E, also lowered the levels of liver function parameters such as AST, ALT, and GGT in the serum but did not distinctly regulate bile acids.

UDCA has long been used for the treatment of PBC and non-alcoholic steatohepatitis (NASH) because it decreases liver biochemical parameters, including alkaline phosphatase and GGT, and delays the need for liver transplantation^15,16^. However, the benefits of UDCA for liver disease have remained controversial. Thus, Ratziu *et al*.^17^ have reported that even at high doses of UDCA (25–35 mg/kg/day), administered for 12 months, no therapeutic effects were observed in patients with NASH. On the other hand, Dufour *et al*.^18^ have reported a positive effect after UDCA treatment for 2 years.

In our study, UDCA treatment effectively reduced liver enzymes (AST, ALT, and GGT). However, the AST and ALT levels were not significantly reduced in the UDCA group at week 8 because one subject appeared to have had dramatic increases in serum parameters, including cholesterol levels, and showed contrasting results in metabolomic profiling (data not shown). UDCA also significantly reduced the circulating miR-122 level, which has been used as an early diagnostic biomarker of liver damage^6^. As shown in Table 1, the miR-122 level started to decrease at week 4 of UDCA administration. Vitamin E treatment also decreased the miR-122 level at week 4, but the decrease was not significant. UDCA is known to regulate the levels of hydrophobic bile acids, such as LCA and DCA conjugates, by increasing the total bile acid pool size, thereby lowering the cytotoxicity^15^. We confirmed this effect of UDCA by our targeted metabolomic analysis (Fig. 2), which we used to measure bile acids at the level of enterohepatic circulation. As expected, UDCA and its conjugates, TUDCA and GUDCA, gradually increased, whereas the DCA level decreased after 8 weeks of treatment. Meanwhile, vitamin E did not cause any typical alterations in the levels of bile acids. These data reveal that UDCA controls hydrophobic bile acids and miR-122 in liver dysfunction patients.

Global metabolomics has recently been used to identify a panel of metabolic biomarkers that help distinguish between the stages of liver disease, including NAFLD, NASH, and cirrhosis^19–21^. Despite numerous studies supporting the efficacy of UDCA in NASH, metabolomic profiling has not been previously conducted to elucidate its protective mechanisms.

Using metabolomic analysis, we investigated the UDCA capacity as an antioxidant. The elevated levels of uremic toxins (hippuric acid and *p*-cresol) and oxidative stress markers (3-nitrotyrosine and 4-HNE GSH) were controlled in the NAFLD patients after 8 weeks of treatment with UDCA. 3-Nitrotyrosine is a cytotoxic molecule, reducing the cell survival, and 4-HNE GSH is a product of 4-HNE conjugation with GSH, resulting from lipid peroxidation^22^. UDCA increased the *N*-acetylcysteine content, resulting in increased synthesis of GSH and ascorbic acid. UDCA has been reported to activate nuclear factor (erythroid-derived 2)-like 2 (NRF2) signalling in PBC patients^23^. As shown in Fig. 4 and Supplementary Tables S2 and S3, some metabolites of the phenylalanine pathway (4-hydroxybenzenesulphonic acid, 4-hydroxyphenylacetic acid, and salicyl alcohol), uremic toxins (hippuric acid and *p*-cresol sulphate), and antioxidants (*N*-acetylcysteine and ascorbic acid) were identified as metabolomic candidates in the UDCA group. In addition, vitamin E reduced tryptophan-originated uremic toxins and lowered some inflammatory lipids. Most regulatory mechanisms of UDCA are related to phenylalanine/tyrosine metabolism, while those of vitamin E are directly related to inflammatory metabolites. The data of metabolomic profiling suggested different regulatory mechanisms for UDCA and vitamin E against liver damage.

Apart from bile acid regulation, imbalanced production of AAAs and BAAs as circulating biomarkers of liver damage has frequently been reported^19,21^. In particular, an increased ratio of AAAs to BAAs has been suggested as a circulating biomarker of NAFLD^21^. Interestingly, several abundant metabolites, distinctively changed in the UDCA group, were associated with the phenylalanine/tyrosine pathway. Most of them were reduced after 8 weeks of treatment. A positive correlation between phenylalanine and ALT has been suggested; however, there is still no adequate evidence^24^. The liver is the predominant site of phenylalanine metabolism; therefore, deterioration of liver function, indicated by abnormally high levels of aminotransaminases (ALT, AST, and GGT), may decrease phenylalanine metabolism, thereby increasing its systemic level.

Changes in AAAs may be associated with the gut microbiome. *N*-acetyltryptophan, 6-hydroxyindole, and 5-hydroxy-6-methoxyindole glucuronide are tryptophan intermediates, well known to be bacteria-derived metabolites. Treatment modalities depend on circulating levels of tryptophan, phenylalanine, and tyrosine^25^. Therefore, we examined the fluctuations in microbial composition during drug treatment. It is well known that gut bacteria secrete small molecules to communicate with the host; bacteria-produced EVs contain various molecules, including proteins, nucleic acids, and lipids, and easily travel within the host body, independent of transporters. EVs are ubiquitously produced by all gram-negative and some gram-positive bacteria, and their presence in biofluids can be used to assess microbial compositions^26–28^. Recently, an EV-based metagenomics study has been used to determine diagnostic biomarkers for pregnancy^27^, atopic dermatitis^26^, and type 2 diabetes^29^.

We found that UDCA reduced the levels of *Lactobacillus* and *Bifidobacterium* after 8 weeks of treatment. *Lactobacillus-*, *Bifidobacterium-*, *Bacteroides-*, and *Clostridium*-rich microbiomes have been found to interrupt the synthesis of gut bacterial metabolites^30^. UDCA reduced the levels of phenylalanine/tyrosine and tryptophan metabolites as shown in Fig. 4 and Supplementary Tables S2 and S3. However, it remains unclear whether the microbiota directly control the synthesis of these aromatic amino acids or inhibit their metabolism. Vitamin E treatment reduced the abundance of *Bacteroidetes* and decreased the ratio of *Bacteroidetes/Firmicutes* at week 8 (Fig. 6E). Although the relationship between vitamin E and *Bacteroidetes* has not been demonstrated yet, a recent study has reported that vitamin E supplementation effectively lowers the inflammatory response and *Bacteroidetes* abundance in infants with iron deficiency^31^.

A study combining metabolomics and metagenomic analysis has suggested that administration of Tempol (4-hydroxy-2,2,6,6-tetramethylpiperidin-1-oxyl) inhibits farnesoid X receptor (FXR) signalling, resulting in bile acid dysregulation, and reduces the *Lactobacillus* abundance and bile salt hydrolase (BSH) activity in obese animals^12^. BSH, produced by *Lactobacillus* and *Bifidobacterium*, catalyses the deconjugation of glycine or taurine derivatives of bile acids, especially DCA and LCA^32^. The mechanism of BSH in bile regulation is a matter of controversy. Theoretically, BSH dehydrolases bile acids, leading to their recycling through enterohepatic circulation, which is beneficial to the host; however, unconjugated forms are more hydrophobic and require active excretion^32^. Although our results are in partial agreement with those of previous reports, a further study is required to compare the change in microbial composition at the faecal level. Taken together, we suggest that intestinal host–microbiome interactions play diverse roles in the pathophysiological process and may exert therapeutic effects.

In summary, this was the first study to apply a systematic approach, using targeted and global metabolomics and metagenomic analysis, to elucidate the underlying mechanisms of UDCA effects in patients with liver dysfunction. The findings suggest that UDCA effectively improves the liver function through various mechanisms, including bile acid regulation, metabolism regulation, and relevant microbiome remodelling, as well as by exerting antioxidant effects similar to those of vitamin E.

## Methods

This study was registered on the ClinicalTrials.gov website under registration no. NCT03000218 (date of registration: 21/12/2016). The study was performed in accordance with the Declaration of Helsinki and Korean Good Clinical Practice guidelines. Participants from this study were originally enrolled as part of a study aiming to evaluate pharmacokinetics and biomarkers after UDCA administration to overweight subjects with liver problems. The study protocol was reviewed and approved by the Institutional Review Board of the Seoul National University Bundang Hospital, Seongnam, Korea (IRB number: B-1602/336-006). All subjects provided written informed consent prior to participating in the study.

### Study design

Male subjects, aged between 20 and 45 years, were recruited, and the specific inclusion criteria were slightly raised ALT levels (40–200 IU/L) and a body mass index of 25–30 kg/m^2^. Of the 20 subjects enrolled, 10 were randomly assigned to receive UDCA (URSA®, Daewoong Pharmaceutical Co., Ltd.) treatment (300 mg, twice daily), and 10 were assigned to receive vitamin E (GRANDPHEROL, Yuhan Co., Ltd.) treatment (400 IU, twice daily). Vitamin E served as a positive control, known to enhance antioxidant defence mechanisms. Both drugs were administered orally for 8 weeks. During this period, one subject withdrew from the UDCA group for a personal reason after 4 weeks and therefore, was excluded from data analysis.

### Sample preparation

Blood (serum and plasma) and urine samples were collected at pre-dose, week 4, and week 8. Blood samples (6 mL) were separately drawn into heparinised tubes and serum separating tubes. The samples were centrifuged (4 °C, 1,800 × *g*, 8 min), and three plasma and serum aliquots (0.8 mL) were stored in Eppendorf tubes at −70 °C until analysis. Urine samples were collected in 15-mL Falcon tubes and stored at −70 °C until analysis. Frozen samples were thawed at 4 °C, and 50 µL of each sample was diluted with 80% methanol (high-performance liquid chromatography grade, Millipore, Bedford, MA, USA) for plasma and 10% methanol for urine samples. The diluted samples were vortexed for 10 min and centrifuged at 18,341 × *g* for 20 min at 4 °C. The supernatants were subjected to global metabolomic analysis, and pooled samples were used as quality control samples.

### Bile acid profiling

A total of 15 bile acids were quantified using the Biocrates® bile acids kit (Biocrates Life Science AG, Innsbruck, Austria) using a liquid chromatography–tandem mass spectrometry (MS/MS) system, allowing concurrent high-throughput detection and quantification of metabolites in plasma samples. The detailed protocol is provided in Supplementary Information.

### Untargeted metabolomics

Each sample (5 μL) was loaded onto an ACQUITY UPLC BEH C18 column (1.7 μm, 2.1 × 100 mm; Waters Corp., Milford, MA, USA), held at 40 °C, and eluted with 0.1% formic acid and 20 mM ammonium formate in water (solvent A) and 0.1% formic acid in methanol (solvent B) at a constant flow rate of 0.4 mL/min and the following gradient conditions: 0–0.1 min, 2% B; 0.1–13 min, 2–98% B; 13–15 min, 98% B; 15–15.1 min, 98–2% B; and 15.1–17 min, 2% B. Subsequently, the eluate was analysed using an Agilent 6530 QTOF mass spectrometer (Agilent Technologies). The overall quality of the analysis procedure was monitored using repeat extracts of a pooled plasma or urine sample (Fig. 2). Urine samples were analysed in a negative electrospray ionisation (ESI) mode, and plasma samples were analysed in a positive ESI mode. The intensity of each ion was normalised, scaled, z-transformed, and aligned according to the retention time using the Mass Hunter Profinder B.08.00 and Mass Profiler Professional (MPP) software package B.14.9 (Agilent Technologies) to generate a normalised data matrix consisting of the retention time, *m/z* value, and peak area. Subsequently, PCA was performed using the MPP software for both positive and negative ESI datasets to determine sample clustering and distinguishing ions (filtered by P < 0.05, adjusted for FDR between pre-dose and week 8).

### Identification of metabolites

The resulting metabolites were identified using the ID Browser tool in the MPP software package B.14.9, a widely used annotation module, matching an accurate mass with the mass tolerance, retention time, and isotope pattern in the following databases: the Agilent METLIN database, Human Metabolome Database, Kyoto Encyclopaedia of Genes and Genomes, and BioCyc. Moreover, if there were commercially available authentic standards, we compared the MS/MS patterns and chromatographic retention times of resultant metabolites.

### miRNA measurement

Total RNA was extracted from serum (100 μL) for quantitative analysis of miRNAs using an miRNeasy mini kit (Qiagen, Hilden, Germany) according to the manufacturer’s recommendations. A synthetic miRNA from *Caenorhabditis elegans* was included in the sample after its homogenisation in the QIAzol lysis reagent (Qiagen) to validate the RNA extraction efficiency. cDNA was prepared using a miScript reverse transcription kit II (Qiagen). The polymerase chain reaction (PCR; 20 µL) contained 10 μL of the miScript SYBR Green PCR master mix (Qiagen), 4 μL of nuclease-free water, 2 μL of 10 × miScript primer assay, 2 μL of 10 × miScript universal primer, and 2 μL of the cDNA template. Amplification was performed using a CFX96 real-time system (BioRad, Hercules, CA, USA) under the following conditions: initial denaturation at 95 °C for 15 min, followed by 40 cycles of denaturation at 94 °C for 15 s, annealing at 55 °C for 30 s, and elongation at 70 °C for 30 s. The total exosome isolation reagent (Invitrogen, Carlsbad, CA, USA) was used for exosome isolation from the serum according to the manufacturer’s instructions.

### Metagenomic analysis

The experimental procedure is detailed in the Supplementary Information.

### Statistical analysis

Statistical analysis was performed using GraphPad Prism version 7 (GraphPad Software, Inc., La Jolla, CA, USA). For comparison of paired samples, a non-parametric equivalent Wilcoxon signed-rank test was used. Repeat-measures analysis of variance was used to compare group means with an equal number of sample points. P-values < 0.05 were considered to indicate statistical significance.

### Data availability

All data generated or analysed during this study are included in this published article and its Supplementary Information files.

## Electronic supplementary material

Supplementary Information
