# Author Correction: Ursodeoxycholic acid improves liver function via phenylalanine/tyrosine pathway and microbiome remodelling in patients with liver dysfunction

**DOI:** 10.1038/s41598-019-53737-7

**Published:** 2019-11-13

**Authors:** Da Jung Kim, Seonghae Yoon, Sang Chun Ji, Jinho Yang, Yoon-Keun Kim, SeungHwan Lee, Kyung-Sang Yu, In-Jin Jang, Jae-Yong Chung, Joo-Youn Cho

**Affiliations:** 1 Department of Clinical Pharmacology and Therapeutics, Seoul National University College of Medicine and Hospital, Seoul, Korea; 2 Department of Clinical Pharmacology and Therapeutics, Seoul National University College of Medicine and Bundang Hospital, Seongnam, Korea; 3 MD Healthcare Inc., Seoul, Korea; 4 Metabolomics Medical Research Center, Seoul National University College of Medicine, Seoul, Korea

Correction to: *Scientific Reports* 10.1038/s41598-018-30349-1, published online 08 August 2018

The Article contains an error in the legend of Table 1.

“Data are the mean ± standard deviation. ^†^P < 0.05 (Friedman test) between pre-dose and week 4; ^‡^P < 0.05 between pre-dose and week 8. ns, not significant.”

should read:

“Data are the mean ± standard error of mean. ^†^P < 0.05 (Wilcoxon signed-rank test) between pre-dose and week 4; ^‡^P < 0.05 between pre-dose and week 8. ns, not significant.”

