# Supplementary material for: Ursodeoxycholic acid improves liver function via phenylalanine/tyrosine pathway and microbiome remodelling in patients with liver dysfunction
Source: Sci Rep. 2018 Aug 8;8:11874. doi: 10.1038/s41598-018-30349-1 (PMC6082879; doi:10.1038/s41598-018-30349-1)
Supplement: Supplementary file 1 — Supplementary Information [file 41598_2018_30349_MOESM1_ESM.pdf]

Supplementary Information

Ursodeoxycholic acid improves liver function via phenylalanine/tyrosine pathway and microbiome remodelling in patients with liver dysfunction

Da Jung Kim[1], Seonghae Yoon[2], Sang-Chun Ji[1], Jinho Yang[3], Yoon-Keun Kim[3], SeungHwan Lee[1], Kyung-Sang Yu[1], In-Jin Jang[1], Jae-Yong Chung[2]* & Joo-Youn Cho[1]*

[1]Department of Clinical Pharmacology and Therapeutics, Seoul National University College of Medicine and Hospital, Seoul, Korea. [2]Department of Clinical Pharmacology and Therapeutics, Seoul National University College of Medicine and Bundang Hospital, Seongnam, Korea. [3]Institute of MD Healthcare, Seoul, Korea.

Table of Contents

Supplementary Materials and Methods………………………………………………..3

Bile acid profiling……………………………………………………………………3

Metagenomic analysis…………………………………………………………………3

Extracellular vesicle isolation and DNA extraction from human urine samples……………………..3

Bacterial metagenomic analysis using EV DNA from human urine samples…………………………4

Analysis of bacterial composition in the microbiota…………………………………………4

Supplementary Fig. S1. Chao1 score and beta-diversity comparisons of microbiomes collected before and after UDCA and vitamin E treatments.……………………………………………..6

Supplementary Fig. S2. Taxonomic profiles of microbiome samples collected before and after treatment……………………………………………………………………………..8

Supplementary Table S1. Urinary metabolites identified by global metabolomic analysis……9

Supplementary Table S2. Plasma metabolites identified by global metabolomic analysis……12

Supplementary Materials and Methods

Bile acid profiling

Plasma samples were prepared according to the manufacturer's instructions. For liquid chromatography–tandem mass spectrometry (LC-MS/MS) analysis, 10 μL of a diluted filtrate was injected into an Agilent 1200 series high-performance liquid chromatography (HPLC) system (Agilent Technologies, Santa Clara, CA, USA), consisting of a binary pump (G1312A) and an autosampler (G1367B), with a thermostat (G1330B) set at 10 °C. Separation was achieved on an analytical column using the Biocrates® bile acids (BAs) kit (Biocrates Life Science AG, Innsbruck, Austria), equipped with an AJ0-4287 SecurityGuard™ ULTRA cartridge for a C18 HPLC column (Phenomenex, Torrance, CA, USA). Mobile phase A comprised 10 mM ammonium acetate (NH_4Ac) and 0.015% formic acid in Milli-Q® water, and mobile phase B comprised 10 mM NH_4Ac and 0.015% formic acid in acetonitrile:methanol:Milli-Q® water (65:30:5, v/v/v). An API 4000 QTRAP (Applied Biosystems/MDS Sciex, Foster City, CA, USA), equipped with an electrospray ionisation source, was used for MS analysis. Seventeen BAs were identified and quantified using the LC-MS/MS system (scheduled multiple reaction monitoring). The abundance of each BA was calculated from the area under the curve by normalisation to its respective isotope-labelled internal standard using the Analyst® 1.5.2 software (Applied Biosystems/MDS Sciex). Calibration curves, quality controls, and samples were evaluated using the MetIDQ™ software (Biocrates). Outliers were removedif samples from more than 50% of the subjects showed values below the limit of detection. A heatmap view was generated using MetaboAnalyst; each concentration was transformed into a log scale for visualisation.

Metagenomic analysis

Extracellular vesicle isolation and DNA extraction from human urine samples

Extracellular vesicles (EVs) from human urine samples were isolated using differential centrifugation at 10,000 × *g* for 10 min at 4 °C. After centrifugation, bacteria and foreign particles were thoroughly

eliminated by sterilising the supernatant by filtration through a 0.22-μm filter. To extract DNA, EVs were boiled for 40 min at 100 °C. To eliminate remaining floating particles and debris, the supernatant was collected after 30 min of centrifugation at 13,000 rpm at 4 °C, and EV DNA was extracted using a PowerSoil DNA isolation kit (MO BIO Laboratories, Inc., Carlsbad, CA, USA) following the standard protocol. DNA was quantified using a QIAxpert system (Qiagen, Germany).

Bacterial metagenomic analysis using EV DNA from human urine samples

Bacterial genomic DNA was amplified with the 16S_V3_F (5′-TCGTCGGCAGCGTCAGATGTGTATAAGAGACAGCCTACGGGNGGCWGCAG-3′) and 16S_V4_R (5′-GTCTCGTGGGCTCGGAGATGTGTATAAGAGACAGGACTACHVGGGTATCTAATCC-3′) primers, which are specific for the V3–V4 hypervariable regions of the 16S rRNA gene. Libraries were constructed using polymerase chain reaction products according to the MiSeq system guide (Illumina, San Diego, CA, USA) and quantified using the QIAxpert system (Qiagen). Amplicons were quantified, pooled at an equimolar ratio, and then sequenced using the MiSeq system (Illumina) according to the manufacturer’s recommendations.

Analysis of bacterial composition of the microbiota

Raw pyrosequencing reads were filtered according to the barcode and primer sequences using the MiSeq system (Illumina). Taxonomic assignment was performed using the profiling program MDx-Pro ver. 1 (MD Healthcare, Korea). This program allows the selection of high-quality sequencing reads after examining the read length (≥ 300 bp) and the quality score (average Phred score ≥ 20). Operational taxonomic units were clustered using the sequence clustering algorithm CD-HIT. Subsequently, taxonomic assignment was carried out using UCLUST and QIIME against the 16S rDNA sequence

database in GreenGenes 8.15.13. Based on sequence similarities, all 16S rDNA sequences were assigned at several taxonomic levels. The bacterial composition at each level was plotted as a stack bar. If clusters could not be assigned at the genus level because of the lack of sequences or redundant sequences in the database, the taxon was assigned at a higher level, which is indicated in parenthesis.

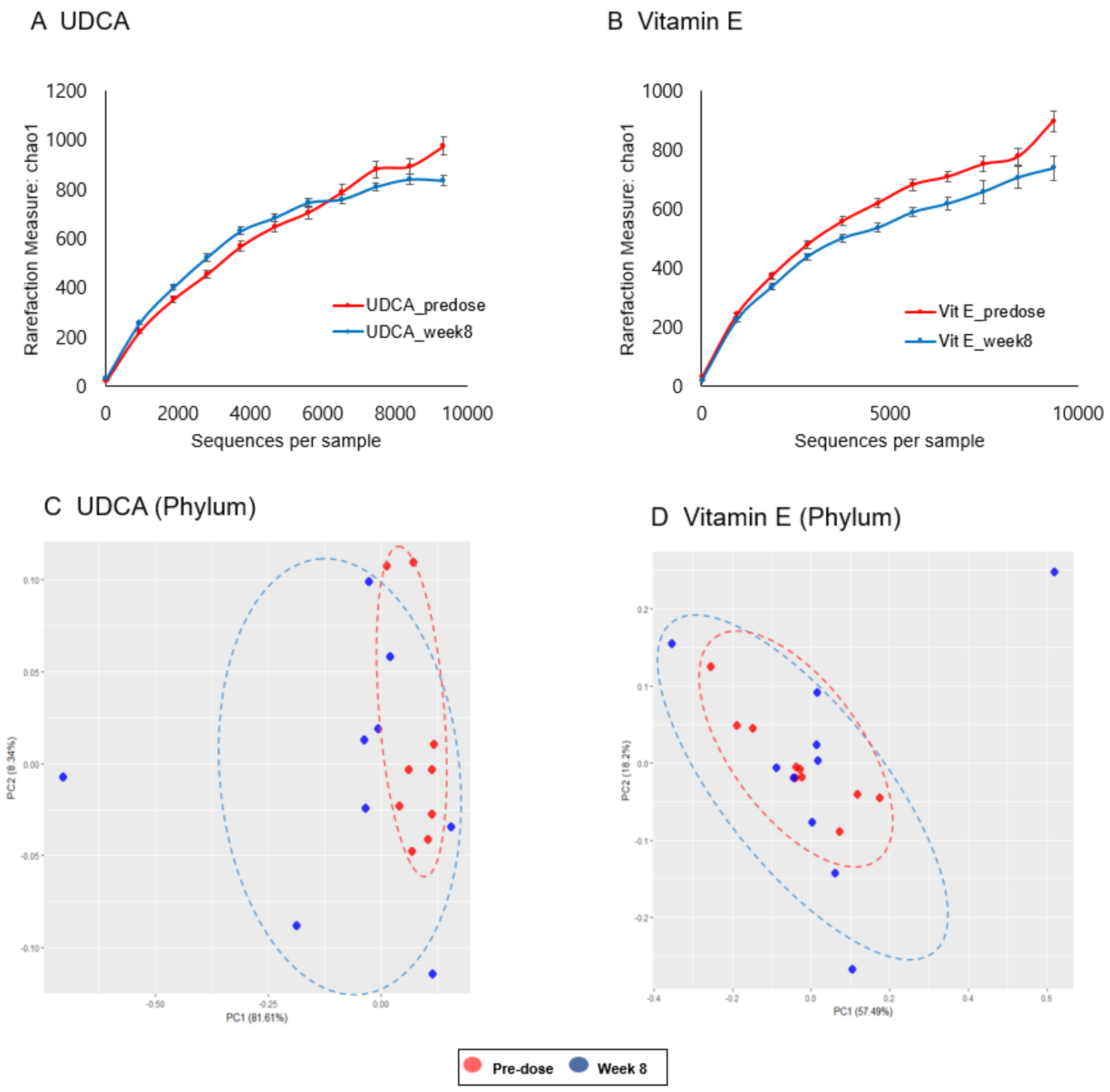

Supplementary Fig. S1. Chao1 score and beta diversity comparisons of microbiomes collected before and after UDCA and vitamin E treatments.

Analysis was performed using sequencing data for the 16S rDNA V3 and V4 regions, with a rarefaction depth of 10,000 reads per sample. Whiskers in the boxplot represent the range of the minimum and maximum alpha diversity values within a population, excluding outliers. Alpha diversity was lower in EV samples collected before the treatment than in those collected after the treatment. (A and B) Chao 1 scores of the UDCA and vitamin E groups, respectively. The results are expressed as the mean ±

standard deviation. (C and D) Principal coordinate analysis (PCoA) of samples from the UDCA and vitamin E treatment groups at the phylum level, respectively. The proportion of variance explained by each principal coordinate axis is denoted in the corresponding axis label. PCoA showed clear separation between samples collected before and after each treatment. Red and blue circles indicate samples collected at pre-dose and week 8, respectively.

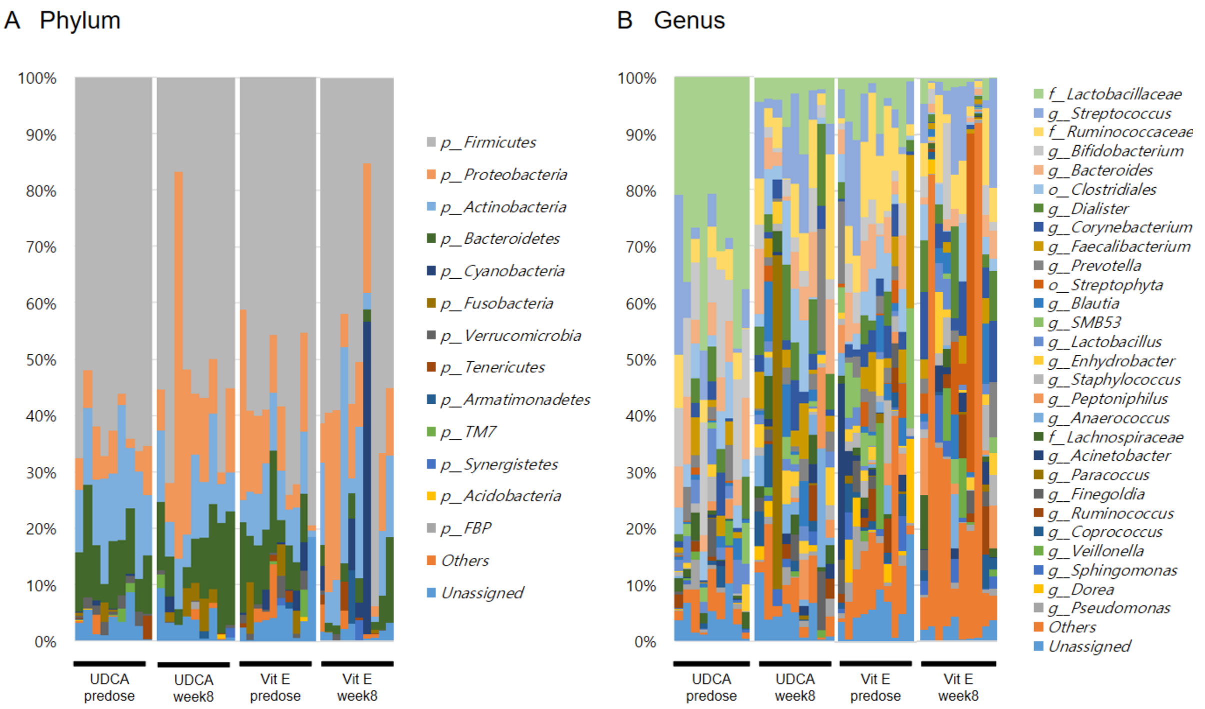

Supplementary Fig. S2. Taxonomic profiles of microbiomes collected before and after treatment.

Analysis was performed using sequencing data for the 16S rDNA V3 and V4 regions, with a rarefaction depth of 10,000 reads per sample. (A) Plots of the relative taxon abundance for samples collected before and after UDCA or vitamin E treatment, summarised at the phylum level. Individual samples are presented along the horizontal axis, and relative taxon frequencies are denoted by the vertical axis. (B) Plots of the relative taxon abundance for samples collected before and after UDCA or vitamin E treatment, summarised at the genus level. Individual samples are presented along the horizontal axis, and relative taxon frequencies is denoted by the vertical axis.

Supplementary Table S1. Urinary metabolites identified by global metabolomic analysis in a negative ESI mode.

tR (min)	Mass (*m/z*)	Delta Da	Adduct	Chemical formula	Identity	q-Value	Identification	UDCA vs. pre-dose	Vitamin E vs. pre-dose
0.7100	197.0722	−0.0034	[M+H]⁻	$C_9H_{11}NO_4$	3-(3,4-Dihydroxyphenyl)-DL-alanine	0.0216	MS/MS	2.65	
1.0550	233.0329	0.0029	[M+H]⁻	$C_8H_{11}NO_5S$	Dopamine 3-*O*-sulphate	0.0216	MS/MS		−2.69
1.1500	255.9875	0.0014	[M+H]⁻	$C_6H_8O_9S$	Ascorbate sulphate	0.0216	MS/MS	3.58	3.78
1.4700	165.0620	0.0170	[M+H]⁻	$C_9H_{11}NO_2$	L-Phenylalanine	0.0216	MS/MS	−2.27	
1.8400	168.0372	0.0051	[M+H]⁻	$C_8H_8O_4$	Homogentisic acid	0.0216	MS/MS	−3.26	
2.0600	226.0916	−0.0326	[M+H]⁻	$C_9H_{10}N_2O_5$	3-Nitro-L-tyrosine	0.0256	MS/MS	−2.60	
2.3560	169.0355	0.0020	[M+H]⁻	$C_7H_7NO_4$	Dihydrodipicolinic acid	0.0228	Database		3.81
2.8200	154.0279	−0.0013	[M+H]⁻	$C_7H_6O_4$	2,4-Dihydroxybenzoic acid	0.0216	MS/MS	2.96	3.45
3.5840	194.0654	0.0037	[M+H]⁻	$C_9H_{10}N_2O_3$	Aminohippuric acid	0.0228	Database		−2.76
3.6300	173.9963	0.0024	[M+H]⁻	$C_6H_6O_4S$	4-Hydroxybenzenesulphonic acid	0.0216	MS/MS	−3.28	−2.74
3.7700	247.9936	−0.0104	[M+H]⁻	$C_6H_6N_3O_6S$	Vanillin sulphate	0.0216	MS/MS	3.27	
3.7900	168.0406	0.0017	[M+H]⁻	$C_8H_8O_4$	3,4-Dihydrophenylacetic acid	0.0402	MS/MS	2.54	
3.8000	152.0458	0.0015	[M+H]⁻	$C_8H_8O_3$	4-Hydroxyphenylacetic acid	0.0228	Database	−2.55	−3.41
3.8100	163.0278	0.0025	[M+H]⁻	$C_5H_9NO_3S$	*N*-Acetyl-L-cysteine	0.0216	MS/MS	2.85	
3.8700	166.0664	−0.0034	[M+H]⁻	$C_9H_{10}O_3$	Phenylacetic acid	0.0317	Database	−2.32	

4.0000	339.0924	0.0030	$[M+H]^{-}$	$C_{15}H_{17}NO_8$	5-Hydroxy-6-methoxyindole glucuronide	0.0216	MS/MS	3.83	3.85
4.0200	383.1042	0.0035	$[M+H]^{-}$	$C_{14}H_{17}N_5O_8$	Succinyladenosine	0.0216	MS/MS	3.39	2.95
4.1000	213.0063	0.0033	$[M+H]^{-}$	$C_8H_7NO_4S$	Indoxylsulphuric acid	0.0114	MS/MS		−2.20
4.1300	152.0461	0.0012	$[M+H]^{-}$	$C_8H_8O_3$	*m*-Hydroxyphenylacetic acid	0.0402	MS/MS	2.28	
4.2000	138.0286	0.0031	$[M+H]^{-}$	$C_7H_6O_3$	Salicylic acid	0.0256	MS/MS	2.86	
4.2010	212.0654	0.0031	$[M+H]^{-}$	$C_{10}H_{12}O_5$	Vanillactic acid	0.0216	Database		2.40
4.3400	133.0512	0.0016	$[M+H]^{-}$	C_8H_7NO	6-Hydroxyindole	0.0216	MS/MS	−2.92	2.95
4.4500	159.0874	0.0021	$[M+H]^{-}$	$C_7H_{13}NO_3$	Acetyl-DL-valine	0.0228	MS/MS	3.40	
4.9600	179.0336	0.0246	$[M+H]^{-}$	$C_9H_9NO_3$	Hippuric acid	0.0228	MS/MS	−2.89	−3.07
5.1100	168.0411	0.0012	$[M+H]^{-}$	$C_8H_8O_4$	Vanillic acid	0.0216	MS/MS	−2.94	
5.2300	146.0669	−0.0090	$[M+H]^{-}$	$C_6H_{10}O_4$	Monomethyl glutaric acid	0.0216	MS/MS	2.38	
5.2400	124.0504	0.0020	$[M+H]^{-}$	$C_7H_8O_2$	Salicyl alcohol	0.0216	MS/MS	−3.22	−3.44
5.3600	218.1137	−0.0082	$[M+H]^{-}$	$C_{12}H_{14}N_2O_2$	Melatonin	0.0216	MS/MS	2.60	
5.5700	122.0362	0.0006	$[M+H]^{-}$	$C_7H_6O_2$	Hydroxybenzaldehyde	0.0216	MS/MS	−3.35	
5.5800	207.0858	0.0037	$[M+H]^{-}$	$C_{11}H_{13}NO_3$	*N*-Acetyl-L-phenylalanine	0.0216	MS/MS	−2.91	
5.7900	209.0675	0.0013	$[M+H]^{-}$	$C_{10}H_{11}NO_4$	Hydroxyphenylacetylglycin e	0.0228	Database	2.99	
5.8300	188.0114	0.0029	$[M+H]^{-}$	$C_7H_8O_4S$	*p*-Cresol sulphate	0.0216	MS/MS	−2.86	−2.60
5.9200	284.0464	0.0432	$[M+H]^{-}$	$C_{13}H_{16}O_7$	*p*-Cresol glucuronide	0.0216	MS/MS	3.31	2.92
6.1300	166.0741	−0.0111	$[M+H]^{-}$	$C_9H_{10}O_3$	(±)-α-Methoxyphenylacetic acid	0.0216	MS/MS	−3.01	

6.7900	203.1135	−0.0553	$[M+H]^-$	$C_{11}H_9NO_3$	3-(3-Indolyl)-2-oxopropanoic acid	0.0256	MS/MS		
6.8600	246.0973	0.0031	$[M+H]^-$	$C_{13}H_{14}N_2O_3$	*N*-Acetyltryptophan	0.0256	MS/MS	2.78	
6.8800	174.0732	0.0160	$[M+H]^-$	$C_8H_{14}O_4$	Diethyl succinate	0.0216	MS/MS	−3.01	−2.26
7.0600	218.0209	0.0040	$[M+H]^-$	$C_8H_{10}O_5S$	Tyrosol-4-sulphate	0.0216	Database	−3.31	−2.68
8.4000	336.1342	0.1322	$[M+H]^-$	$C_{21}H_{36}O_3$	Pregnanetriol	0.0256	Database	−2.53	
9.3009	384.1518	0.0037	$[M+CH_3COOH]^-$	$C_{13}H_{25}O_7P$	DHAP(10:0)	0.0228	Database		−3.82
10.0899	440.0588	−0.0002	$[M+CH_3COOH]^-$	$C_{10}H_{13}N_4O_{10}P$	Urate D-ribonucleotide	0.0216	Database		3.83
10.4479	370.1841	−0.0027	$[M+H]^-$	$C_{19}H_{30}O_5S$	5α-Dihydrotestosterone sulphate	0.0114	MS/MS		3.10

Statistically significant ($p < 0.05$) differences are shown for week 8 vs. pre-dose. *q*-Values were adjusted for the false discovery rate ($q < 0.05$). MS/MS indicates that the fragmentation of each identified metabolite was confirmed using the standard compound. ‘Database’ indicates that the fragmentation of each identified metabolite was searched in databases (HMDB and METLIN).

Supplementary Table S2. Plasma metabolites identified by global metabolomic analysis in a positive ESI mode.

tR (min)	Mass (*m/z*)	Delta Da	Adduct	Chemical formula	Identity	q-Value	Identification	UDCA vs. pre-dose	Vitamin E vs. pre-dose
6.0050	180.0877	0.0022	$[M+H]^+$	$C_9H_{12}N_2O_2$	5-Hydroxykynurenamine	0.0006	MS/MS	−3.25	
5.8220	265.1421	−0.0230	$[M+NH_4+H]^{2+}$	$C_{13}H_{16}N_2O_3$	6-Hydroxymelatonin	0.0094	Database	−2.94	
9.0901	340.2337	0.0044	$[M+Na]^+$	$C_{21}H_{34}O_2$	Epipregnanolone	0.0045	MS/MS	3.04	
11.2852	381.2626	0.0018	$[M+H]^+$	$C_{18}H_{40}NO_5P$	Sphinganine phosphate	0.0101	MS/MS	2.93	
8.6141	577.1917	0.4083	$[M+H]^+$	$C_{21}H_{34}N_3O_{10}SF_3$	4-Hydroxynonenal glutathione	0.0359	MS/MS	−2.70	
0.6800	145.1108	0.0471	$[M+H]^+$	$C_7H_{19}N_3$	Spermidine	0.0011	MS/MS		3.03
0.6690	117.0778	0.0012	$[M+H]^+$	$C_5H_{11}NO_2$	Betaine	0.0056	Database		2.80
0.6870	271.1662	−0.0018	$[M+H]^+$	$C_{11}H_{21}N_5O_3$	Prolylarginine	0.0201	Database		−2.55
7.2300	278.1638	−0.0008	$[M+H]^+$	$C_{15}H_{22}N_2O_3$	Phenylalanyl-isoleucine	0.0203	Database		−2.54
10.5849	272.2127	0.0013	$[M+H]^+$	$C_{19}H_{28}O$	5a-Androst-3-en-17-one	0.0011	MS/MS		3.00
11.3268	330.2434	−0.0028	$[M+H]^+$	$C_{18}H_{34}O_5$	(+)-9,10,18-Trihydroxy-12Z-octadecenoic acid	0.0398	Database		2.41
11.7858	122.0727	0.0005	$[M+H]^+$	$C_8H_{10}O$	2,6-Dimethylphenol	0.0070	Database		2.76
12.3178	253.2370	0.0040	$[M+H]^+$	$C_{16}H_{31}NO$	Palmitoleamide	0.0398	MS/MS		2.41
12.4588	297.2655	0.0015	$[M+H]^+$	$C_{18}H_{35}NO_2$	3-Ketosphingosine	0.0170	Database		−2.59

13.9647	299.2849	−0.0025	$[M+H]^+$	$C_{18}H_{37}NO_2$	Sphingosine	0.0100	MS/MS	−2.70

Statistically significant ($p < 0.05$) differences are shown for week 8 vs. pre-dose. q-Values were adjusted for the false discovery rate ($q < 0.05$). MS/MS indicates that the fragmentation of each identified metabolite was confirmed using the standard compound. ‘Database’ indicates that the fragmentation of each identified metabolite was searched in databases (HMDB and METLIN).
